# Interactions Between Live Attenuated Influenza Vaccine and the Nasopharyngeal Microbiota among Children Aged 24-59 Months in The Gambia: A Phase Iv Open Label, Randomised Controlled Clinical Trial

**DOI:** 10.1016/j.lanmic.2024.100971

**Published:** 2025-01-17

**Authors:** Chikondi Peno, Ya Jankey Jagne, Melanie Clerc, Carlos Balcazar Lopez, Edwin P. Armitage, Hadijatou Sallah, Sainabou Drammeh, Elina Senghore, Gabriel Goderski, Sophie van Tol, Adam Meijer, Alicia Ruiz-Rodriguez, Wouter de Steenhuijsen Piters, Emma de Koff, Sheikh Jarju, Benjamin B. Lindsey, Janko Camara, Sulayman Bah, Nuredin I. Mohammed, Beate Kampmann, Ed Clarke, David H. Dockrell, Thushan I. de Silva, Debby Bogaert

**Affiliations:** a Centre for Inflammation Research, Queen’s Medical Research Institute, University of Edinburgh, Edinburgh, United Kingdom; b Vaccines and Immunity Theme, Medical Research Council Unit The Gambia at the London School of Hygiene and Tropical Medicine, Banjul, The Gambia; c Centre for Infectious Disease Research, Diagnostics and Laboratory Surveillance, National Institute for Public Health and the Environment, Bilthoven, The Netherlands; d The Vaccine Centre, Faculty of Infectious and Tropical Diseases, The London School of Hygiene and Tropical Medicine, London, United Kingdom; e Centre for International Child Health, Section of Paediatrics, Department of Medicine, Imperial College London, London, United Kingdom; f Division of Clinical Medicine, School of Medicine and Population Health, The University of Sheffield, Sheffield, United Kingdom; g Department of Paediatric Immunology and Infectious Diseases, Wilhelmina Children’s Hospital/ University Medical Center Utrecht, Utrecht, The Netherlands; h Department of Epidemiology of Microbial Diseases, Yale School of Public Health, Connecticut, New Haven, USA

**Keywords:** Live attenuated influenza vaccine, nasopharyngeal microbiota, vaccine immunogenicity-bacterial microbiota interactions, viral-bacterial microbiota interaction

## Abstract

**Background:**

Live attenuated influenza vaccine (LAIV) alters nasopharyngeal microbiota in adults. LAIV immunogenicity varies across populations for reasons poorly understood, but may be linked to the microbiome. We investigated the impact of LAIV on nasopharyngeal microbiota, and the impact of nasopharyngeal microbiota on LAIV immunogenicity in Gambian children.

**Methods:**

We conducted a phase IV open label randomised controlled trial in clinically well children aged 24-59 months in The Gambia (NCT02972957). Participants were randomised 2:1, stratified by sex, to receive trivalent LAIV at recruitment (day 0 (D0), LAIV group) or after follow-up (day 21, control group) using pre-prepared computer-generated sequence. Primary outcomes were impact of LAIV on microbial communities as determined by 16S rRNA sequencing, and impact of microbiota on LAIV-induced mucosal IgA responses. Respiratory viral co-infection (asymptomatic) at recruitment, and year of were included as covariates. The trial is closed for recruitment.

**Findings:**

Between February 8 and April 12, 2017, and January 15 and March 28, 2018, 343 children were screened for eligibility. We analysed samples from 213 children receiving LAIV and 108 controls. Although we did not observe an independent impact of LAIV on microbial community structure at day 7 or day 21, we observed an effect of LAIV depending on year of recruitment. LAIV impacted microbiome structure in 2018 (Effect size(R^2^)=1.97%,95%CI; 0.85%-5.94%, p=0.04) but not in 2017 (R^2^=1.23%, 95%CI; 0.49%-4.46%, p=0.09). We also observed an impact of viral co-infection at baseline on day 7 microbiota regardless of year (R^2^ =1.01%,95%CI;0.28%-3.01%, p=0.02). D0 microbiota had no impact on mucosal IgA responses to LAIV

**Interpretation:**

The impact of LAIV on nasopharyngeal microbiota in children is modest and temporary. Our data shows that the impact of LAIV on nasopharyngeal microbiota in children is modest and temporary, therefore suggests that LAIV can be used as an intervention to curb Influenza infections in children from LMICs without causing long-lasting perturbations on the mucosa microbiota. However, nasopharyngeal microbiota at the time of vaccination might not explain inter-individual variability observed in LAIV-induced IgA responses.

**Funding:**

The Wellcome Trust, UK National Institute for Health Research, Chief Scientist Office Scotland.

## Introduction

Respiratory tract infections are a leading cause of morbidity in children particularly those in low and middle income countries (LMICs).^1^ It is estimated that each year approximately 870,000 hospitalisations in children are associated with seasonal influenza infections with the highest burden experienced in developing countries.^2^ Influenza vaccines such as live attenuated influenza vaccines (LAIV) are key interventions in preventing seasonal Influenza infections. LAIV is highly protective against Influenza virus infection in young children^3^, and could therefore be used to prevent or reduce the burden of Influenza infections in children and adults from low-income settings.

The human upper respiratory tract is a dynamic and complex environment, and a habitat to both commensal and potentially pathogenic organisms. Bacteria and viruses co-colonise this niche and interact with the host and other microbes. As an intranasal live viral vaccine, LAIV infects nasal epithelial cells and parallels some aspects of the wild-type influenza host-pathogen interactions, including increasing the bacterial burden of potential pathogens such as *Staphylococcus aureus* and *Streptococcus pneumoniae*. ^4,5^ In this cohort, we have recently shown that LAIV modestly increased carriage density of *S. pneumoniae* for up to 21 days, akin to effects caused by asymptomatic respiratory viral infection.^5^ However, the immediate effects of LAIV on the overall nasopharyngeal microbiome in children remains unknown.

Previous studies across different populations show that LAIV immunogenicity and efficacy varies among individuals and across populations.^6,7^ Emerging evidence supports the role of microbiota in shaping host immune responses, including those induced by vaccination as well as infection at the time of exposure.^8^ Most evidence to date demonstrates that gut microbiota influences parenteral and oral vaccine-induced immune responses,^9^ but little is known about how the human nasopharyngeal microbiota might influence responses to vaccines delivered to the upper respiratory tract. Evidence using germ-free mice indicate that the upper respiratory tract microbiota can calibrate the thresholds of activation for innate antiviral responses via macrophage interferon-signaling pathways, thereby potentially influencing virus-specific T-cell and nasal IgA responses.^10^ Investigating similar relationships between nasopharyngeal microbiota and LAIV-induced immune responses could provide a better understanding on the basis for the poor performance of mucosal vaccines in resource-limited settings and may inform future microbiota-targeted interventions to improve mucosal vaccine responses.

Here we conducted a phase IV longitudinal randomized controlled clinical trial to explore interactions between LAIV and nasopharyngeal microbiota in Gambian children. Specifically, we investigated whether intranasal immunisation with LAIV results in alterations of nasopharyngeal microbiota and whether the composition of the nasopharyngeal microbiota at the time of vaccination is associated with LAIV-induced immune responses.

## Methods

### Study design and study participants

We conducted a prospective longitudinal randomised phase IV clinical trial (NCT02972957) in Sukuta, a peri-urban community in The Gambia. ^5,6^ The study was conducted as part of the NASSIMUNE study secondary outcomes (NCT02972957). Participants were children aged 24 to 59 months, with no underlying illness and no history of respiratory illness for at least 14 days before the day of recruitment (full inclusion and exclusion criteria in appendix, pp 3-4). Study participants were recruited between two years; between February 8 and April 12 in 2017, and between January 15 and March 28 in 2018. Participants were randomised to receive LAIV on the day of recruitment (day 0; D0, LAIV group) or delayed vaccination at day 21 (D21, control group).^5^

The study was approved by The Gambia Government and UK Medical Research Council joint ethics committee and the Medicines Control Agency of The Gambia (Reference; SCC1502). A parent provided written or thumb printed informed consent for their children to participate. For parents who were not English literate, an informed consent discussion was conducted in a local language in the presence of an impartial witness. The impartial witness signed to confirm completeness of the consent provided. The trial protocol is provided in appendix.

### Randomisation and masking

Children were randomly assigned 1:1:1, with two groups to receive trivalent LAIV on the day of enrolment (day 0 LAIV group) or delayed until day 21 (control group). Randomisation was performed in permutation blocks of fifteen with stratification by sex, using sealed opaque envelopes and a computer-generated randomisation sequence pre-prepared by an independent statistician not involved in the rest of the study. The investigator team was blinded to block size and randomisation sequence to avoid allocation bias. Group allocation was revealed upon opening the envelope, cross-checked, and co-signed by a second individual. As an open-label study, from this point on the investigator team were aware of group allocation. Due to the need to carry out LAIV immune response assays on samples in the LAIV group only, group allocation was also revealed to the local laboratory team after completion of recruitment. The external laboratory where nasopharyngeal microbiome characterisation was executed was blinded to group allocation until analysis.

### Procedures

Northern hemisphere Russian-backbone trivalent LAIV (Nasovac-S, Serum Institute of India, Pune, India) was used in both years, containing 2009 pandemic H1N1 (A/17/California/2009/38 in 2017 and A/17/New York/15/5364 in 2018), H3N2 (A/17/Hong Kong/2014/8296) and influenza B-Victoria (B/Texas/02/2013) viruses.

Nasopharyngeal swabs (NPS) were collected from all study participants at baseline (day 0, D0), day 7 (D7) and D21 using flocked paediatric swabs (FLOQSwabs, Copan, CA, USA) and stored in RNAprotect® (Qiagen, United Kingdom). Samples were processed within 4 hours of collection and stored at -70°C until further processing. In addition, participants randomised to receive LAIV at D0 had oral fluid from the buccal cavity obtained using swabs (Oracol Plus; Worcester, UK) at D0 and D21, along with whole blood samples for T-cell response assays. To minimize study visits for participant and number of blood collections, participants receiving LAIV at D0 were further randomised 1:1 to have additional whole blood sample collected at either D7 post LAIV for *ex vivo* Tfh response assays described in this study, or day 2 (blood used for other study objectives).

Bacterial DNA was extracted from the NPS as previously described. ^11^Amplicon libraries were generated by PCR using F515/R806 primers targeting the 16S rRNA hypervariable (V4) region and sequenced using Illumina Miseq sequencing platform (Illumina Inc, USA).. Sequencing data were processed to obtain operational taxonomic units (OTUs) using a bioinformatics pipeline as previously described (Appendix).^12^ To avoid multiple OTUs with identical annotations, OTUs were named using taxonomic annotations combined with a rank number based on average relative abundance of each OTU across the entire dataset. Only OTUs present at >0.1% relative abundance in at least two samples were used in the analyses.^13^ We used the *decontam* package (combined method) to identify and remove environmental contaminating OTUs using default parameters.^14^

All samples collected at D0 were screened for respiratory viral presence using a multiplex real time PCR (RT-PCR) as previously described.^15^ Influenza A, influenza B, Respiratory Syncytial Virus (RSV) A, RSV B, human parainfluenza viruses (HPIV) 1-4, human metapneumovirus, adenovirus, seasonal coronaviruses (CoV; 229E, OC43, NL63) and human rhinovirus were included in the panel.^15^

Haemagglutinin inhibition (HAI) titers, influenza haemagglutinin-specific IgA and T-cell responses were measured as previously described.^6^ HAI assays were performed on vaccine strain haemagglutinin-matched and neuraminidase-matched viruses. Seroconversion to LAIV was defined as a four-fold or greater increase in HAI titers (to ≥1:40) from D0 to D21. ^6^Mucosal influenza-specific IgA was measured in oral fluid samples at baseline and D21 post-LAIV using a protein microarray as previously described,^16,17^ with the percentage Surfact-Amps-20 in the blocking, washing and incubation buffer increased from 0.05% to 5% to prevent background staining with oral specimens. Total IgA was quantified using an ELISA and influenza-specific IgA expressed as a ratio of influenza HA1-specific IgA/total IgA as previously described,^17^ with a two-fold or greater increase from D0 to D21 considered a significant response.

Quantification of CD4+ and CD8+ T-cell responses was done by stimulating 200µl whole blood for 18 hours with overlapping 15-18-mer peptide pools (2µg/ml) covering vaccine-matched Matrix and Nucleoprotein (MNP; 47 and 68 peptides respectively), haemagglutinin 1 (HA1; 74 peptides) and haemagglutinin 3 (HA3; 74 peptides) as previously described (Appendix pp5).^18^A two-fold or higher increase from D0 to D21 samples was considered a significant response.^6^ We measured *ex vivo* peripheral T-follicular helper-like (Tfh-like) cells as a proxy for germinal centre activity by flow cytometry. A volume of 200µl of whole blood was used for the staining. Additional unstained and FMO controls for ICOS and CXCR5 were included. CXCR5 bv421 was added to the tubes and incubated for 15 minutes at 37°C and 5% CO_2_. Surface staining was done using CD4, CD8, CD45R0 and ICOS. followed by a 15-minute incubation at room temperature in the dark (Appendix pp 5, 10). Cells were then lysed with 2ml of 1 x BD FACS solution and incubated at room temperature for 10 minutes in the dark. FACS buffer was added to the cells and centrifuged at 1800rpm for 5 minutes. Cells were resuspended in 250µl FACS buffer, and stored at 4°C in dark until acquisition. Cells were analysed on a LSR Fortessa flow cytometer. Tfh-like cells were defined as CD4^+^ T cells expressing CD45RO, CXCR5 and ICOS (Appendix pp 19). Tfh-like CD4^+^ cell responders were defined as ‘High’ when fold increase from D0 to D7 was equal to or above the median fold change in the whole dataset.

### Outcomes

The primary outcomes were 1. the impact of LAIV on D7 and D21 nasopharyngeal microbiome profiles, and 2. the association between the nasopharyngeal microbiome at time of vaccination on LAIV-induced mucosal IgA responses. Secondary outcomes were the impact of the nasopharyngeal microbiome on seroconversion using a haemagglutinin inhibition assay, Influenza A Matrix and Nucleoprotein CD4^+^ T-cell responses (MNP) and induction of e*x vivo* peripheral Tfh-like CD4^+^ cells. An exploratory outcome was the impact of D0 asymptomatic respiratory viral infection on the nasopharyngeal microbiota post-LAIV to investigate their interaction as previous described.^5,12^ Post-hoc analyses were impact of LAIV on D7 nasopharyngeal microbiome stratified by year of recruitment.

### Statistical analysis

Sample size calculations were based on the potential association between baseline nasopharyngeal microbiota and induction of mucosal influenza-specific IgA, as a representative LAIV-induced immune response. Power calculations were performed using a sample size microbiome studies shiny application (https://fedematt.shinyapps.io/shinyMB) aiming to detect at least two-fold differences in the 50 most abundant bacteria after correction for multiple testing.^19^ At an alpha of 0.05 (two-sided), 82 subjects per group would provide 80% power to detect differences between IgA responders and non-responders. A sample size of n=103 per group (total n=206 receiving LAIV) was selected with 50% expected to mount an IgA response (2-fold increase from D0 to D21) and accounting for potential dropouts (up to 20%), which still provided ≥80% power to address our research question on the impact of the microbiome on immunogenicity. Children completing their day 21 visit with available samples were included in the final modified intention-to-treat analysis.

Permutational analysis of variance (PERMANOVA) tests were used to study the association between overall microbial community composition (response variable), LAIV administration and other covariates of interest or known to influencing microbial composition (appendix pp 19), over the whole study period, and at D7 and D21 post-LAIV (explanatory variables; adonis-function, vegan R-Vegan package). We performed nonparametric bootstrapping to infer 95% confidence intervals of the effect sizes by generating 1000 OTU matrices based on randomly sampled taxa in the original OTU matrix with replacement. We then fitted PERMANOVA models on each resampled OTU matrix and calculated confidence intervals of the estimated effect sizes as the 2.5% and 97.5% percentiles.

Microbiota profiles were identified using unsupervised average linkage hierarchical clustering based on the Bray-Curtis matrix previously described (appendix pp 7, pp 20).^12^ Only clusters with 10 or more samples were considered for further statistical analyses. Biomarker OTUs for the microbiota clusters (profiles) were determined using Random Forest models based on the *VSURF package*. Biomarker taxa ascertainment for microbiome clusters were based on relative abundances (appendix pp 20-21). Chi-square tests or Fisher’s exact tests were used to assess associations between microbiota profiles and LAIV vaccination. We used Fisher’s exact tests when more than 20% of cells in the contingency table had frequencies of less than five. When the expected cells of the contingency table had five or more, and no cell had expected values of less than one, we performed the Chi-square test.

Differentially abundant taxa between LAIV recipients and controls were identified using Microbiome Multivariable Associations with Linear Models (MaAsLin2) using default parameters (OTU minimum prevalence of 10%, false discovery rate (FDR) ≤0.25, obtained using Benjamin-Hochberg (BH)).^20^

PERMANOVA tests were used to identify associations between overall microbial community composition and immunogenicity outcomes. Chi-square tests or Fisher’s exact tests where appropriate were used to assess associations between D0 microbiota profiles and LAIV immunogenicity outcomes. Linear models were performed to study associations between biomarker taxa relative abundances and LAIV immunogenicity outcomes. The obtained p-values were adjusted for multiple testing using the Benjamin-Hochberg method to obtain q-values. In addition, tests Wilcoxon rank-sum tests were performed as non-parametric tests to compliment results from linear models in cases where model assessments were poor (appendix pp 26-29).

All statistical analyses were done using R software version 4.0.1, R Studio 1.3.959.

### Role of the funding source

The funder had no role in study design, data collection, data analysis, data interpretation or writing of the report.

## Results

**Figure 1:**
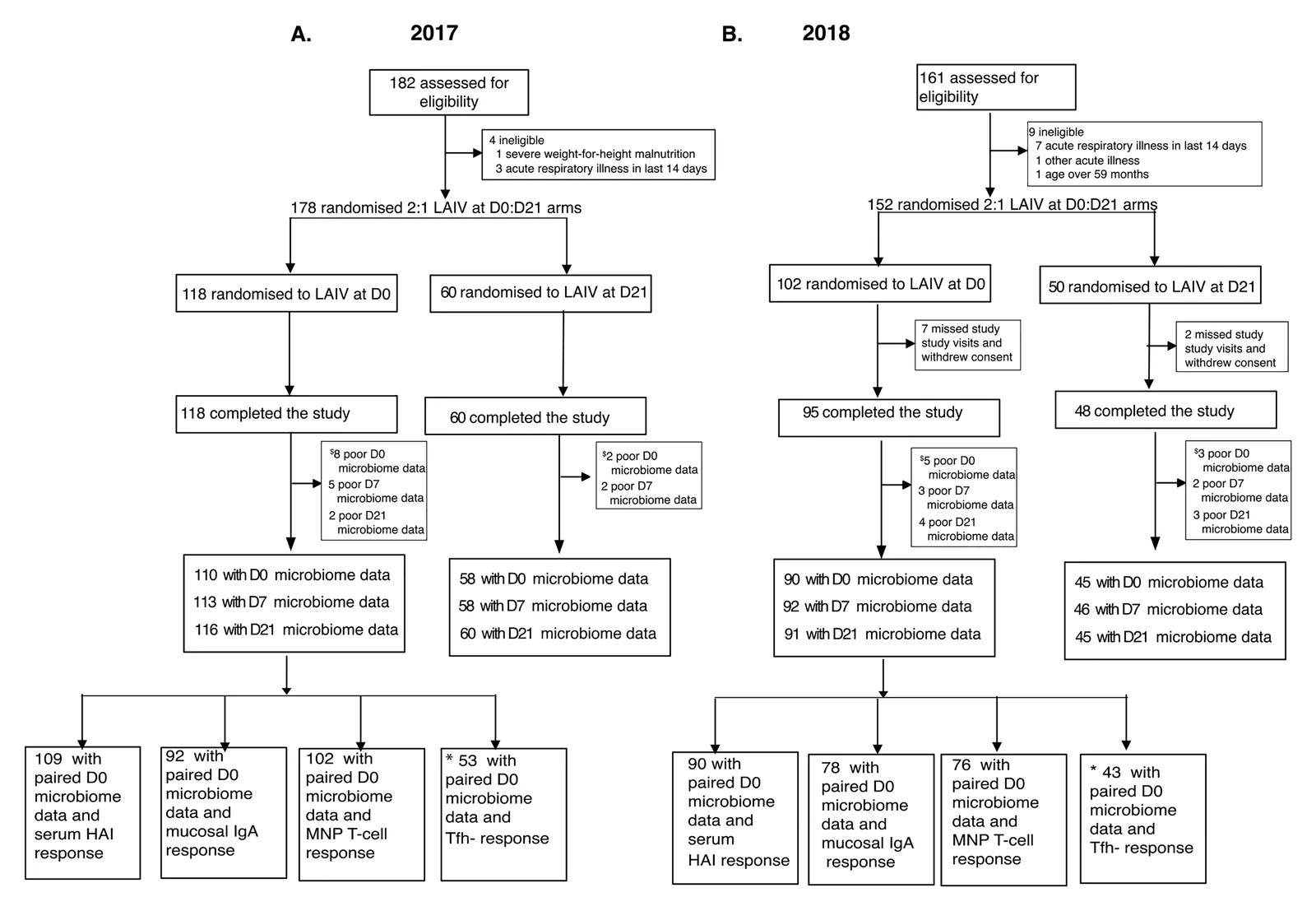
Study overview detailing participants recruitment, retention and final number of samples included in the analysis. The study was a randomised controlled phase 4 clinical trial with registration number NCT02972957. **A)** recruitment in 2017 **B)** recruitment in 2018. LAIV=live attenuated influenza vaccine, D0=Day 0 (Baseline), D21= Day 21 study Visit. MNP= Influenza A matrix and nucleoprotein, Tfh=T follicular helper cell to LAIV. ^$^Poor microbiome data was defined as either having low DNA concentration on 16s qPCR/low read count following sequencing. * Ex vivo Tfh-responses at D0, D7 and D21 were measured in 107 of the children (approximately half the cohort) in the LAIV group to minimise study visits and bleeds as per the overall trial study design.

Between February 8 and April 20, 2017, and January 15 and 28, March 2018, 343 healthy children were recruited and assessed for eligibility according to the study inclusion criteria (Figure 1). Of these, 220 children were randomised to the LAIV group and 110 to the control group. All children recruited in 2017 completed the study. In 2018, 95 (93%) children randomised to the LAIV group and 48 (96%) randomised to the control group completed the study (Figure 1). All study visits were within protocol-defined windows (+1 day for D2 visit, +7 days for D7 visit, +7 days for D21 visit) with 96% occurring on the exact visit day. In the LAIV group, 199 (93%) children had paired data for assessing the impact of D0 microbiome on seroconversion, 170 (80%) for influenza-specific mucosal IgA response, 178 (84%) for MNP-specific CD4+ T-cell responses and 96 (45%) for Tfh-like responses (Figure 1).

Baseline characteristics are summarised in Table 1. Asymptomatic viral infections were detected in 67/205 (33%) children in the LAIV group and 41/104 (39%) in the control group. Rhinoviruses were the most prevalent viruses found in all study groups, representing 75% of all viruses detected in the LAIV group and 61% in the control group (Table 1).

**Figure 2:**
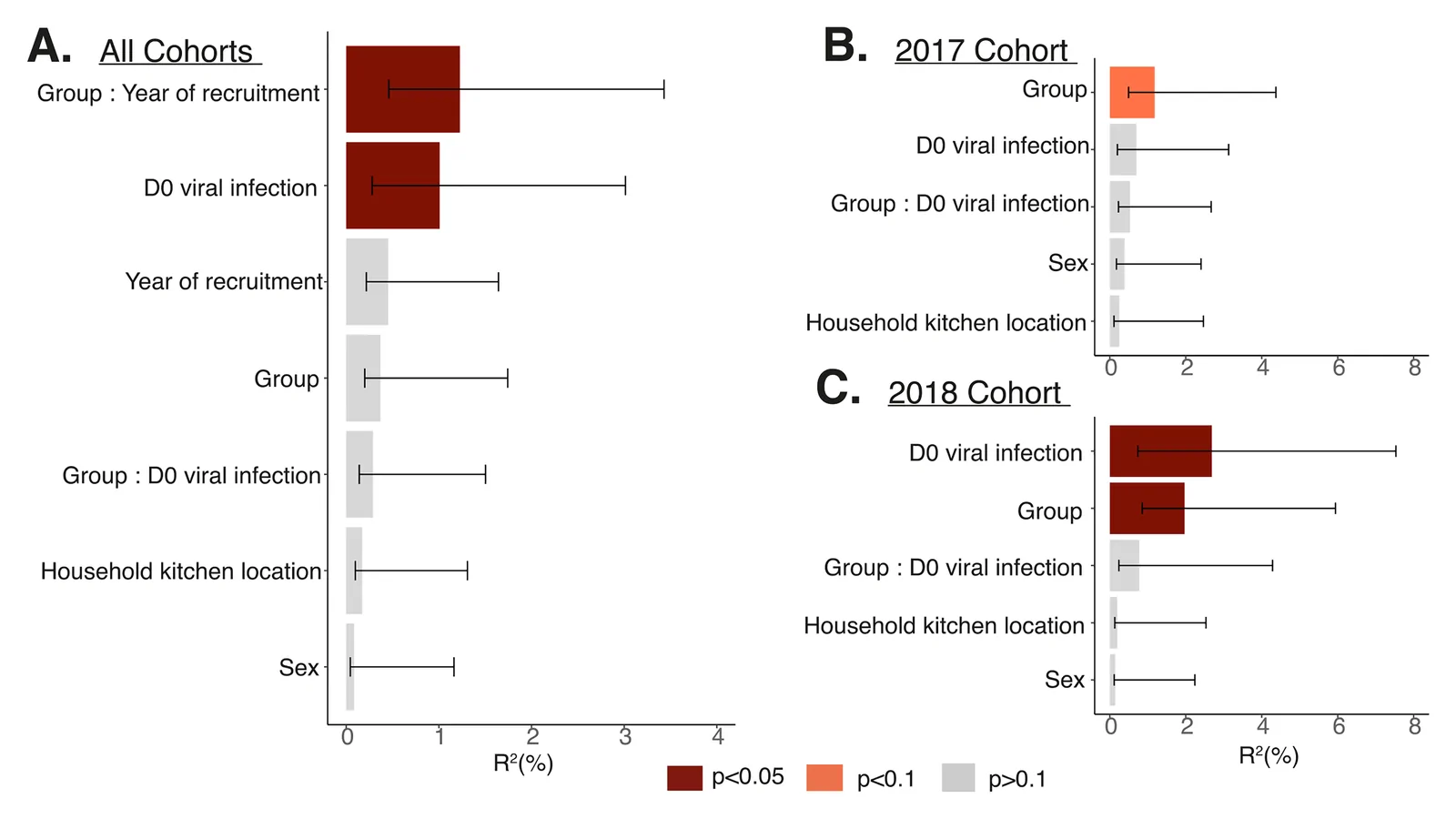
Factors associated with nasopharyngeal microbial community composition at day 7 following LAIV administration. A. nasopharyngeal microbial community composition at day 7 and stratified by year of recruitment with B. Recruitment in 2017 and C. Recruitment in 2018. P values and accompanying effect size (R^2^) obtained using PERMANOVA tests. Dark red= p-value < 0.05, Orange =p-value > 0.05 & < 0.1 (modest significance), Grey = p-value > 0.1 (not significant). Stress =0.18.

First, a PERMANOVA model were fitted to study the impact of LAIV on microbial community structure longitudinally, across the whole study period, and did not observe any impact of LAIV on microbial community structure (R^2^=0.24%, 95%CI; 0.13%-0.89%, p=0.23, appendix pp 11). Furthermore, we assessed microbiota stability over time, by quantifying changes in Bray-Curtis dissimilarity between paired subsequent timepoints per participant, and observed a modest but not significant microbiota instability in the LAIV group between D0-D7 (appendix pp 19). Next, we used a multivariable PERMANOVA model to compare microbiota communities between groups at D7 and D21, adjusting for covariates that significantly or modestly were associated with D0 microbial community composition, such as household cooking location and year of recruitment (as different vaccine formulations were used) (p<0.10, appendix pp 20), or factors that were reported previously to affect microbial community composition following LAIV i.e. presence of asymptomatic respiratory virus at baseline.^5,12^ The PERMANOVA models were further adjusted for sex (variable used for stratification during block randomisation) and included interactions between a) LAIV and year of recruitment, and b) LAIV and presence of baseline asymptomatic viruses. Although we did not find an independent impact of LAIV alone on microbial community structure at day 7 (R^2^ =0.36%,95%CI; 0.20%-1.74%, p=0.34 Figure 2A), we did observe a significant effect for the interaction term including year of recruitment and LAIV on D7 microbial community structure (R^2^ =1.23%,95%CI; 0.46%-3.43%, p=0.01, Figure 2A). In addition, we observed a significant impact of baseline viruses on D7 microbial community structure (R^2^ =1.01%,95%CI; 0.28%-3.01%, p=0.02, Figure 2A). LAIV nor any of the other covariates or interaction terms impacted the microbial community composition at day 21 (appendix pp 11).

To investigate the significant interaction between LAIV and year of recruitment, we performed a stratified analysis, fitting a PERMANOVA model for each year of recruitment (2018 or 2017). This post-hoc analysis showed a significant impact of LAIV on D7 microbial community structure in 2018 (R^2^=1.97%,95%CI; 0.85%-5.94%, p=0.04, Figure 2C) though only a modest and non-significant effect in 2017 (R^2^=1.23%, 95%CI; 0.49%-4.46%, p=0.09, Figure 2B). Interestingly, the post-hoc analysis also showed that the impact of baseline asymptomatic viruses was only observed in 2018 (R^2^=2.68%,95%CI; 0.74%-7.53%, p=0.01) but not in 2017 (R^2^ =0.7%,95%CI; 0.19%-3.11%, p=0.32, Figure 2B). The interaction term between LAIV and presence of baseline asymptomatic viruses was not significant in the main model (R^2^=0.28%,95%CI; 0.13%-0.89%, p=0.47, Figure 2A) nor in the per year stratified models (Figure 2B & 2C), suggesting that the impact of presence of baseline asymptomatic viruses on microbial community structure was independent of LAIV. In the LAIV group, LAIV shedding did not impact D7 or D21 microbial community composition (appendix pp 12-13).

Hierarchical clustering performed on all samples regardless of timepoint (n=927) identified 17 distinct microbiota profiles, of which 6 included 10 or more samples and were used for further statistical analyses (appendix pp 20-21). These microbiota profiles were dominated by either *Dolosigranulum (2)/C. propinquum (4)* (found in 392 samples), *Haemophilus (3)* (n=176), *Moraxella* (n=155), O*rnithobacterium (7*) (n=115), *Streptococcus (5*) (n=45), or *C. propinquum (4) alone* (n=23) (appendix pp 20). The microbiota profiles were equally represented in both the LAIV group and control groups at baseline, except for the *Streptococcus*-enriched profile, of which we excluded from the subsequent analyses (p=0.01,Table 2). At D7, Moraxella-dominated profiles became underrepresented in LAIV recipients compared to controls (RR (95%CI), 0.77(0.58-1.01), p=0.04, Table 2, appendix pp 22). No significant associations between LAIV and microbiota profiles were observed at D21 (Table 2). In addition, the effect of LAIV on taxa level, adjusting for year of recruitment, baseline viral co-infection and household cooking location, showed only three taxa that were less abundant in the LAIV group compared to controls at D7, namely *Neisseriaceae* (44), *Prevotella spp* (181), and the low abundant taxa *Rubellimicrobium (231)* (appendix pp 13), and none that was increased in abundance in the LAIV group. At D21, we did not observe any differences in microbial taxa between the LAIV group and controls (appendix pp 14).

**Figure 3:**
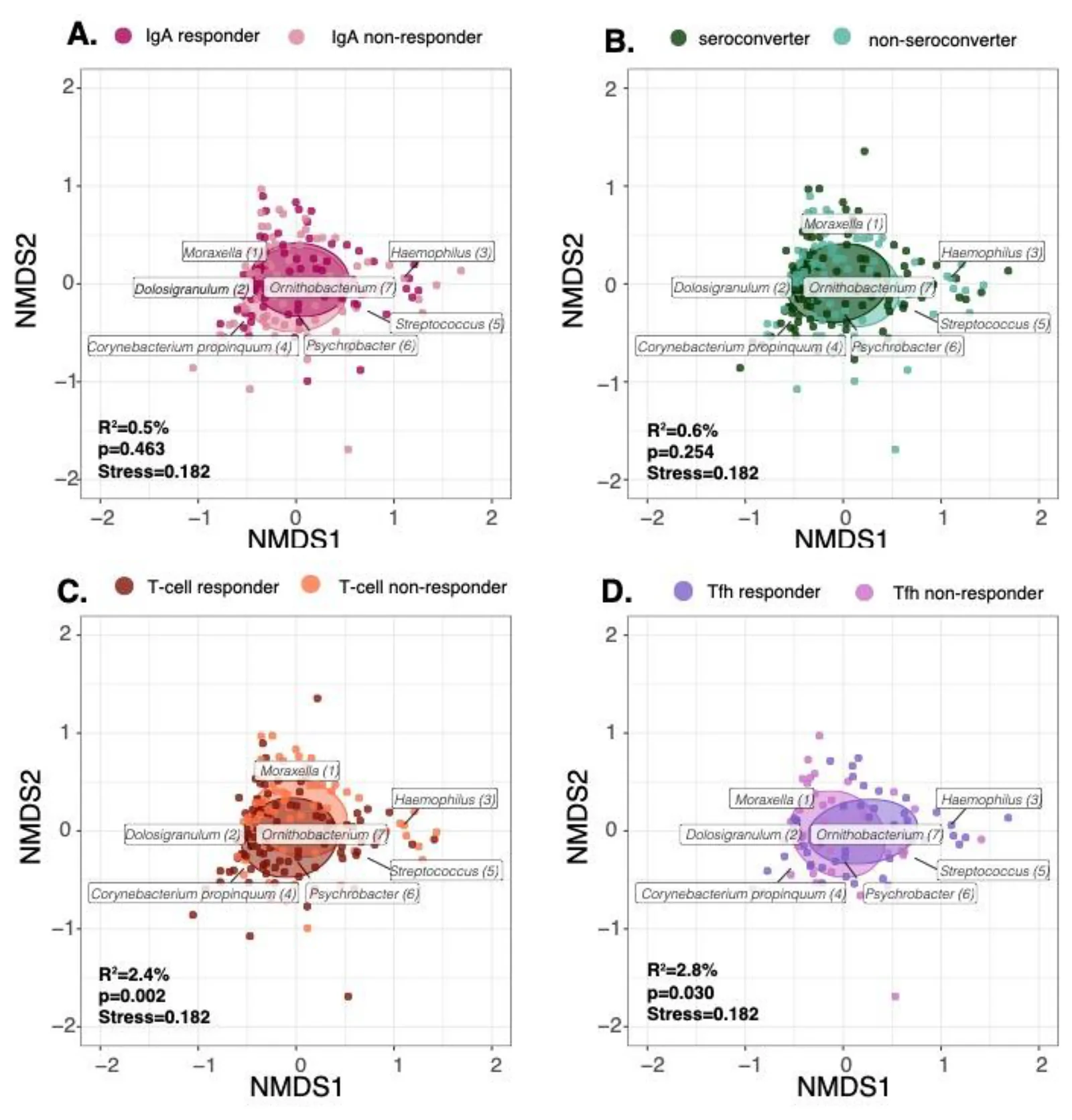
Associations between nasopharyngeal microbiota at day 0 and immune responses to LAIV. Non-metric dimensional scaling (NMDS) plots visualising associations between microbial community composition and immune responses. Projected taxa are biomarker taxa of the microbiota profiles identified by hierarchical clustering. Each data point represents the microbial community composition of a single sample at the time of LAIV receipt. A) IgA response between D0 and D21 B) Seroconversion between D0 and D21 C) Influenza A matrix nucleoprotein (MNP) CD4+ T cell response between D0 and D21 D) T follicular helper-like cell response at D7. R^2^= percentage of variance explained by immune variable, calculated by multivariate permutational multivariate analysis of variance (PERMANOVA) test, adjusting for any co-variates associated with both the D0 microbiome and the immune response to LAIV. NMDS also depicts biomarker taxa in relation with the ordination plot. Stress values for the ordinations are also reported, indicating the goodness of fit of the data points following dimension reduction (<0.1= excellent, <0.2=good, >0.2=poor). LAIV=live attenuated influenza vaccine.

Next, we investigated if the microbiome at time of vaccination influenced LAIV immunogenicity in the LAIV group. A total of 106/199 (53%) seroconverted to either pH1N1 (n=24, 12%), H3N2 (n=49, 25%), or B/Victoria strain (n=58, 29%), which we defined as ‘any seroconversion’ in the subsequent analyses (appendix pp 14). A two-fold increase or more in influenza-specific mucosal IgA response between D0 and D21 to at least one antigen was seen in 74/170 (44%) children, with 38 (22%) responding to pH1N1, 27 (16%) to H3N2 and 56 (33%) to B/Victoria (appendix pp 14). A two-fold increase or greater in intracellular IFN-g or IL-2 CD4^+^ T-cell responses to influenza A MNP peptides from baseline to D21 was observed in 56% (100/178) of children. An increase in *ex vivo* peripheral Tfh-like cells was seen between D0 and D7 (p=0.01; appendix pp 23) but not between D0 and D21 (p=0.22, appendix pp 23). Therefore Tfh-like responders were classified as those with values equal to or above the median D0-D7 fold change. The Tfh cell responses were not correlated with IgA responses to any LAIV strains (appendix pp 23). Using PERMANOVA tests, we found that the D0 microbial community composition was not associated with IgA response (R^2^=0.5%, 95%CI; 0.23%-2.49%, p=0.46, Figure 3A, appendix pp 14-15) or seroconversion (R^2^=0.6%,95%CI; 0.28%-2.55%, p=0.25, Figure 3B, appendix pp 14-15). However, we did observe a significant association between D0 microbial community composition and MNP CD4+ T-cell response (R^2^=2.3%,95%CI; 1.12%-5.21%, p=0.01, Figure 3C, appendix pp 14-15), as well as *ex vivo* peripheral Tfh-like cell response (R^2^=3.1%,95%CI; 1.07%-9.38% p=0.03, Figure 3D, appendix pp 14-15). When investigating IgA responses, the number of LAIV strains shed was also impacted by D0 microbial community composition (appendix pp 15-16). All LAIV immune responses were not influenced by nutrition status at the time of recruitment (appendix pp 16).In addition, D0 microbiota composition was not impacted by nutrition status (appendix 16).

We next investigated whether specific microbiota profiles or taxa were responsible for the differences in immune responses observed. We found that having a *Dolosigranulum (2)/Corynebacterium (4)*-dominated microbiota profiles or higher abundances of these taxa at D0 were associated with MNP CD4+ T-cell responses (RR (95%CI), 1.21(0.93-1.58), appendix pp 16-17, and q=0.02 for *Dolosigranulum*, and q=0.05 for *Corynebacterium*, appendix pp 16;pp-24), while *Moraxella (1)*-enriched microbiota profiles or higher abundances were negatively associated with these MNP CD4+ T-cell responses (RR (95%CI),0.59 (0.36-0.94), q=0.01, appendix pp 16;pp 24). Although microbiota profile analyses also showed that a *Dolosigranulum (2)/ Corynebacterium (4)*-dominated microbiota profiles were associated with seroconversion (RR (95%CI), 1.35 (1.04-1.75), p=0.03, appendix pp 16-17) and Tfh-like responses (RR (95%CI),1.66(1.17-2.35),p=0.01, appendix pp 16-17), taxa level analysis for *Dolosigranulum* or *Corynebacterium* showed higher abundances seen in those with an immune response but were not statistically significant (appendix pp 17-18;pp 24). Similarly, microbiota profile level analyses showed that *Ornithobacterium (7)*-dominated microbiota profiles (RR (95%CI), 0.39(0.14-1.06), p=0.02 appendix pp 16-17) and higher abundances of *Haemophilus (3)* (q-value=0.07, appendix pp 24) were negatively associated with Tfh-like responses, but could not be confirmed at taxa level or microbiota profile level respectively (appendix pp 17;pp 24).

## Discussion

In this randomised longitudinal controlled trial, we investigated interactions between LAIV and the nasopharyngeal microbiota in influenza vaccine naïve children from The Gambia. We show that LAIV administration in children results in modest, short-lived microbial community perturbations, which are amplified by asymptomatic viral co-infections detected at the time of vaccination. We however also show that the microbial community composition of the nasopharynx at the time of vaccination is significantly associated with LAIV-induced immune responses. To our knowledge, this is the first time these interactions have been explored in children from a low-income setting.

Our first finding concurs with those from a UK human challenge model study, where the effect of LAIV on nasopharyngeal microbiota of adult volunteers was modest and diminished rapidly.^12^ The effect of viral co-infections was similar to that of LAIV, which is consistent with previous studies showing perturbations in respiratory microbiota following respiratory viral infections in both symptomatic and asymptomatic cases.^4,21^ The lack of LAIV effect in 2017 is more puzzling. While it is possible that the higher prevalence of viral co-infection in 2018 may explain the lack of an effect observed in 2017, we also speculated that the improved replicative fitness following replacement of the pH1N1 component in the 2018 formulation as observed previously in this same cohort, might be responsible for the observed year effects, however, our results did not find any effect of LAIV shedding on microbial composition.^5,6^

*Moraxella* dominated profiles were underrepresented in the LAIV group following vaccination. *Moraxella*-dominated profiles have been associated with reduced upper and lower respiratory tract infections. ^22,23^ Although specific mechanisms are not established, *Moraxella*-dominated profiles are thought to portray stable microbiota colonisation, possibly owing to the ability to form biofilms potentially contribute to resilience against viral associated dysbiosis. ^22^ Furthermore, our finding on decreased abundances of rare abundant oral taxa including *Neisseria* spp. following LAIV, was not completely in line with observations from a USA adult study, which found more significant reductions in *Corynebacterium* abundance and increase in *Staphylococcus* and *Bacteroides* following LAIV vaccination.^4^ Differences in vaccine formulations used in our cohort and the US study (Russian backbone vs Ann-Arbor formulations), age of participants (adults vs. children) and geographical region may however help to explain the observed variations.

Interestingly, the microbial community composition at the time of vaccination was significantly associated with adaptive immune responses following LAIV, albeit not with mucosal IgA induction. Especially microbiota profiles enriched with lactic acid producing bacteria *D. pigrum* and *C. propinquum* were consistently influenza-specific MNP CD4+ T-cell responses. This is an interesting finding given that *Dolosigranulum* spp. and *C. propinquum* have also been associated with respiratory health in previous studies.^24^ These results are also in line with previous associations between the predominance of other lactic acid producing bacteria in the gut including *Lactobacillus* spp. immune responses to LAIV.^25^ It is possible that the presence of lactic acid bacteria such as *D. pigrum* and *C. propinquum* found in our study, enhance cellular immune responses to live attenuated vaccines in a similar fashion to those observed at other mucosal sites such as the gut. Nonetheless, more trials and experimental models are needed to ascertain these observations and examine potential mechanisms.

We observed that *Ornithobacterium* and *Haemophilus*-dominated profiles were modestly associated with lower immune responses, particularly for the induction of Tfh-like cells. Children densely colonized with *H. influenza*e or showing *Haemophilus*-dominated microbiota profiles exhibit a systemic immune phenotype characterized by increased pro-inflammatory cytokines such as IL-6 and IL-8 as well as enhanced neutrophil recruitment and activation.^26^ This suggests that children with higher *Haemophilus*-dominated microbiota profiles present with a low-grade inflammatory phenotype which might hamper appropriate vaccine-induced immune responses following an immune challenge.

The reasons why we did not observe any association between nasopharyngeal microbiota and LAIV-induced mucosal IgA are not clear. While this may be explained by only 44% of children showing mucosal IgA response and our study being powered based on having 50% IgA responders, there may be other explanations. A study in UK adults found that recent nasopharyngeal colonisation with *S. pneumoniae* prior to LAIV administration dampens mucosal IgA responses to LAIV ^27^ and over 70% of our participants were colonised with *S. pneumoniae* at baseline.^5^ Several hypotheses have been suggested, including viral replication interference by *S. pneumoniae* through stimulation of TLR, or that *S. pneumoniae* colonisation diminishes immune cell infiltration and antigen-presenting cell activation, affecting downstream immune responses.^27^ This is consistence with findings from the gut where a systems biology approach showed that the absence of Toll-like receptor (TLR) 5, which is a sensor of bacterial flagellin, resulted in reduced serum IgG antibody responses to TIV, hinting at the influence of flagellated gut microbes on antibody responses. It is therefore possible that the higher prevalence of pneumococcal colonisation in our cohort may affect immune responses, however our study lacked the power to study this.

Our study has several strengths. We conducted a longitudinal randomised controlled trial which allowed controlling of potential individual microbial variation. Conducting the study outside the peak of influenza season, careful clinical review before recruitment and molecular screening of influenza strains at baseline, greatly minimised confounding effects of natural influenza infections when investigating the effect of LAIV on nasopharyngeal composition. Measuring the multifaceted responses of LAIV-induced immunity which included T cell responses rather than confined to humoral responses in our study, provides a unique perspective on the importance inter-relationships existing between nasopharyngeal microbiota and induced cellular immune responses.

Our study also has several limitations. Because of the low biomass of respiratory samples used in our study, we used 16S rRNA sequencing to characterize the microbial communities presented in this study, which lacks species and strain-level identification and functional insight. However, our recent report on species level effects of LAIV with a particular focus on *S. pneumoniae*, show similar modest perturbations.^5^ This also highlights the limitation of using 16S rRNA sequencing for Streptococcal species which have a high level of sequence homology (>99%) in this region.^28^ In addition, our results on the impact of nasopharyngeal microbiome on LAIV immunogenicity are based on associations rather than causation, and should be followed by mechanistic studies. However, given the randomised and temporal nature of the study, this observational evidence can be considered strong. We were unable to fully explain the reasons behind differences in microbial composition in participants recruited in 2018 which may confound our comparisons between groups. We excluded participants that did not complete their day 21 visit and all samples with poor quality microbiome data from our analysis which could have possibly introduced selection bias during analysis. Lastly, we considered variable inclusion in our analyses guided by knowledge from previous studies and possible biological hypotheses, this selection criteria would have omitted important confounders that may be unique to this setting. Nonetheless, with the RCT study design used, this could have had minimal impact.

Our findings represent the first data from a low- and middle-income setting, elucidating the effects of administering LAIV in children on nasopharyngeal microbiota perturbations and provides evidence that LAIV has modest and temporary effects in interrupting nasopharyngeal microbial communities, in this age group, adding to findings from a study of UK children reporting the impact of LAIV on major bacterial pathogens using qPCR.^29^ Furthermore, we report for the first time the potential influence of nasopharyngeal microbiota on LAIV-induced immune responses in children. Our results suggests that lactic acid producing bacteria in nasopharyngeal microbiota could be important in achieving robust T-cell responses to mucosal vaccination. The crucial role in protection by T cell-mediated immunity induced through vaccination in influenza infections is recently recognised. ^30^ With limitations seen with strain-specific LAIV formulations, efficacy, such as vaccine mismatch T-cell induced responses could be key in providing protection to against influenza infection even when lower LAIV antibodies are induced. The findings from our study therefore provide potential avenues to research on microbiota-targeted interventions that can boost immunogenicity of LAIV and possibly improve vaccine efficacy and effectiveness. Future studies supplementing higher resolution mechanistic data of lactic acid bacteria such as *Dolosigranulum* and *C. propinquum* are needed to provide functional insights.

## Supplementary Material

Appendix

## Figures and Tables

**Table 1:** Summary of baseline characteristics from participants completing the study

	LAIV (n=213)	Control (n=108)
Age in months (median [IQR])	35.00 [28.00, 42.00]	32.50 [28.00, 40.00]
Sex		
Male	116 (55%)	58 (54%)
Female	97 (46%)	50 (54%)
Baseline asymptomatic virus present*	67 (33%)	41 (39%)
Adenovirus	3 (5%)	3 (7%)
Seasonal coronaviruses (sCoV)	7 (10%)	3 (7%)
Parainfluenza 1	2 (3%)	4 (10%)
Parainfluenza 1 & sCoV	1 (2%)	0 (0%)
Parainfluenza 1 & Rhinovirus	1 (2%)	0 (0%)
Parainfluenza 3	0 (0%)	1 (2%)
Rhinoviruses	50 (75%)	25 (61%)
Rhinovirus & Adenovirus	1 (2%)	3 (7%)
Rhinovirus & sCoV	1 (2%)	1 (2%)
RSV A	1 (2%)	0 (0%)
RSV B	0 (0%)	1 (2%)
Influenza A or B	0 (0%)	0 (0%)
Household kitchen location (%)**	189 (89%)	97 (90%)
Presence of smoker in the house	51 (24%)	27 (25%)
Antibiotic use in the last month before recruitment		
None	204 (96%)	103 (95%)
Beta-lactams	4 (2%)	1 (1%)
Cotrimoxazole	4 (2%)	4 (4%)
Chloramphenicol	0 (0%)	0 (0%)
Other^$^	1 (1%)	0 (0%)
Baseline seropositive (haemagglutinin inhibition titre ≥1:10)		
pH1N1	82 (39%)	-
H3N2	146 (69%)	-
B/Vic	68 (32%)	-

Data are presented as n (%) or median and interquartile range [IQR]

* data not available for 8 participants in the LAIV arm and 4 participants in the control arm. Percentages given for individual, or combinations of viruses detected use the total number of children with asymptomatic viruses detected for each group as the denominator.

$ Antibiotics other than Beta-lactam, Cotrimoxazole, Macrolides, Chloramphenicol.

** Cooking inside (under a roof) compared to cooking outside kitchen (open fire). 99% of children lived in households where wood/charcoal was primary fuel used to cook regardless of location of cooking.LAIV=live attenuated influenza vaccine, RSV = Respiratory Syncytial Virus, sCoV=seasonal Coronavirus. Demographic characteristics did not differ between years of recruitment (Table S2) apart from differences in baseline immune responses have been previously reported ^6^.

**Table 2:** Microbiota profiles in LAIV and Control groups.

DAY 0	LAIV (n=196)	Control (n=102)	RR (95% CI)	p-value
***Dolosigranulum (2) /C. propinquum (4)* (n=122)**	83(42.3%)	39(38.2%)	1.06(0.90-1.25)	0.45
***Moraxella (1)*(n=59)**	35 (17.9%)	24 (23.5%)	0.88(0.70-1.11)	0.26
***Haemophilus (3)*(n=59)**	39 (19.9%)	20 (19.6%)	0.97(0.79-1.20)	0.83
***Ornithobacterium (7)*(n=34)**	23 (11.73%)	11 (10.7%)	1.05(0.83-1.33)	0.85
***Streptococcus (5)*(n=11)**	11 (5.6%)	0 (0%)	1.55(1.43-1.70)	0.02
**DAY 7**				
***Dolosigranulum (2)*** ***/Corynebacterium (4)* (n=139)**	93 (46.7%)	46 (46.5%)	1.01(0.87-1.19)	0.83
***Moraxella (1)*(n=47)**	25 (12.6%)	22 (22.2%)	0.77(0.58-1.01)	0.03
***Haemophilus (3)*(n=51)**	33 (16.6%)	18 (18.2%)	0.99(0.79-1.22)	0.89
***Ornithobacterium (7)*(n=45)**	36 (18.1%)	10 (9.1%)	1.21(1.01-1.44)	0.07
***Streptococcus (5)*(n=16)**	12 (6.0%)	4 (4.0%)	1.13(0.84-1.51)	0.59
**DAY 21**				
***Dolosigranulum (2) /C. propinquum (4)*(n=131)**	91 (44.3%)	40 (38.8%)	1.06 (0.91-1.24)	0.44
***Moraxella (1)*(n=49)**	29 (14.1%)	20 (38.8%)	0.87 (0.68-1.12)	0.23
***Haemophilus (3)*(n=66)**	40 (19.5%)	26 (25.2%)	0.90(0.72-1.11)	0.29
***Ornithobacterium (7)*(n=36)**	26 (12.6%)	10 (9.7%)	1.11 (0.90-1.39)	0.36
***Streptococcus (5)*(n=18)**	16 (7.8%)	2 (1.9%)	1.36(1.13-1.63)	0.04

Shown are number and percentage of participants with microbiota at D7 within each profile. p-values identified by chi-square tests or Fisher’s Exact tests were appropriate. *Only clusters with >10 samples were used for the analysis for statistical power. This excluded the Corynebacterium cluster (see appendix). OR=Odds ratio, CI= 95% confidence interval. LAIV=live attenuated influenza vaccine.

## Data Availability

Sequence data that support the findings of this study can be accessed in the US National Center for Biotechnology Information Sequence Read Archive database under BioProject ID PRJNA1102700 upon publication. De-identified participants metadata will be available upon request to the corresponding author (dboagert@ed.ac.uk) with a signed data access agreement.

## Research in Context

### Evidence before the study

We searched PubMed with the terms: “live attenuated influenza vaccine” AND “microbiome” from database inception up to 16 April 2024. This search identified 8 studies, of which following navigation of the title and the abstract, 4 studies investigated the relationships between LAIV and the nasopharyngeal or nasal microbiome in human subjects. The first study was a longitudinal study involving adult volunteers (n=17) in the US and investigated the effect of LAIV on nasal microbiota and gene expression profiles. This study reported significant changes in the nasal microbial community structure and diversity, with an increase in relative abundances of *Staphylococcus* and *Bacteroides* genera following LAIV administration. The second study investigated changes in the nasal microbiome following LAIV, also in US adults (n=47), and investigated if these changes were associated with LAIV-induced mucosal IgA production. The study reported that LAIV administration induced changes in the microbiome allowing less abundant taxa to colonise the anterior nares and reported that these changes were associated with variations in mucosal Influenza-specific IgA antibody responses. The third study used a human challenge model to study interactions between LAIV, pneumococcal challenge and nasal microbiota in UK adults (n=117). This study reported modest perturbations of nasal microbiota by LAIV, with higher perturbations observed at day 2 (R^2^= 1.7%) and diminishing perturbations over time (R^2^= 1.3% at both days 7 and 29). Furthermore, LAIV impacted the receptiveness of the nasopharyngeal microbiota to pneumococcal colonization. The fourth, was a follow up study in the same human challenge cohort (n=90) investigated the effect of experimentally induced pneumococcal colonisation on mucosal LAIV antibody responses. Mucosal IgA in the nose and IgG in the lung were dampened in the group challenged with pneumococci prior to LAIV. We did not find any study investigating the effect of LAIV on the nasopharyngeal microbiome or other respiratory microbiome niches and/or the influence of nasopharyngeal microbiome on LAIV-induced mucosal responses in children or LMIC populations.

### Added value of this study

This study, to our knowledge, is the first randomised controlled trial to report interactions between LAIV and nasopharyngeal microbiota composition in children and is the first study from an African setting. Our results show that LAIV-induced perturbations on nasopharyngeal microbiota are modest. Additionally, we provide the first evidence that the nasopharyngeal microbiota may impact LAIV induced adaptive immune responses.

### Implications of all available evidence

Our results highlight that LAIV administration in children living in a low- and middle-income country does not result in long-lasting perturbations of the nasopharyngeal microbiota and therefore does not pose concerns as an intervention to curb the burden of Influenza in children in this setting. Importantly, our data suggest an important role for nasopharyngeal microbiota in adaptive immune responses to intranasally administered LAIV. The nasopharyngeal microbiome could therefore represent a key target in improving LAIV immunogenicity to both humoral and cellular responses to LAIV in children.
